# Eco-Anxiety in Higher Education Professionals: Psychological Impacts, Institutional Trust, and Policy Implications

**DOI:** 10.3390/ejihpe16010006

**Published:** 2025-12-29

**Authors:** Sarah Louise Steele

**Affiliations:** 1 School of Health and Social Care, University of Essex, Colchester CO4 3SQ, UK; s.steele@essex.ac.uk; 2 School of Psychology, Arden University, Coventry CV3 4FJ, UK; 3 ThinkLab, University of Cambridge, Cambridge CB2 1TN, UK; 4 Intellectual Forum, Jesus College, Cambridge CB5 8BL, UK; 5 Centre for Research on Social Dynamics and Public Policy (Dondena), Bocconi University, 20136 Milan, Italy

**Keywords:** eco-anxiety, social sustainability, higher education, institutional trust, climate governance, mental health, SDG 13, greenwashing, moral injury, solastalgia

## Abstract

Eco-anxiety—emotional distress arising from awareness of environmental collapse—has become a critical dimension of social sustainability, linking mental well-being, professional functioning, institutional trust, and climate governance. This study investigates how higher education professionals (HEPs) experience and interpret eco-anxiety within their professional contexts, situating it as a lens on institutional legitimacy from the perspective of those who produce, teach, and steward climate knowledge. A cross-sectional mixed-methods survey of 556 HEPs was conducted across a month in 2023, combining an adapted climate anxiety scale with open-ended narratives. Quantitative analyses identified perceived governmental inadequacy as the strongest correlate of climate worry (β = 0.48, *p* < 0.001), accounting for 26% of the variance, whereas institutional inadequacy had a weaker effect. Qualitative findings revealed pervasive emotions of moral injury, solastalgia, and exhaustion when sustainability rhetoric outpaced genuine action, with many respondents describing governmental and institutional “betrayal.” Integrating Cognitive Appraisal Theory with concepts of moral legitimacy, the study conceptualises eco-anxiety as a relational and ethically grounded emotion reflecting the perceived misalignment between knowledge and governance. Addressing it requires transparent climate leadership, participatory governance, and organisational care infrastructures to sustain motivation and trust within universities. Eco-anxiety thus may function not only as a personal pathology but also as a psychosocial response that can illuminate HEPs’ perceptions of institutional misalignment with sustainability commitments, with implications for higher education’s contribution to the Sustainable Development Goals.

## 1. Introduction

Climate change is increasingly recognised not only as an ecological and political emergency, but also as a profound psychological issue and institutional challenge. Awareness of environmental crises, including global heating, biodiversity loss, and transgressions of planetary boundaries, has intensified worldwide (Kurth & Pihkala, 2022). These developments have far-reaching psychological consequences, giving rise to emotions such as fear, grief, anger, and despair (Clayton, 2020; Coffey et al., 2021). Scholars across psychology, education, and sustainability science now identify these climate emotions as critical for understanding both individual and institutional engagement in sustainability transitions (Pihkala, 2020a, 2020b). Among them, eco-anxiety—that is, distress associated with awareness of environmental decline and perceived systemic inadequacy—has emerged as a defining psychosocial phenomenon of the Anthropocene (Verplanken et al., 2020; Hickman et al., 2021; Ogunbode et al., 2022).

Although eco-anxiety is not classified as a clinical disorder, its emotional and functional effects are well-documented. It comprises cognitive appraisals of existential threat, moral dissonance, and helplessness, often linked to perceptions of governmental and institutional failure (Clayton & Karazsia, 2020; Budziszewska & Jonsson, 2022; Wang et al., 2023). Research has connected these emotions to broader issues of trust, legitimacy, and governance, revealing that climate distress is relational, arising not only from environmental threat but also from the erosion of confidence in societal actors expected to respond (Ogunbode et al., 2022; Ojala, 2017). Consequently, eco-anxiety represents a nexus between mental well-being, ethics, and institutional accountability rather than a solely individual psychological response.

### 1.1. Higher Education and the Emotional Dimensions of Sustainability

Understanding eco-anxiety within the higher education sector is crucial for achieving the United Nations Sustainable Development Goals (SDGs), particularly SDG 3 (Good Health and Well-Being), SDG 4 (Quality Education), SDG 13 (Climate Action), and SDG 16 (Peace, Justice, and Strong Institutions). Universities serve as both knowledge producers and societal exemplars; they educate future leaders, inform public policy, and advance sustainability science (Leal Filho et al., 2025). Yet, their sustainability engagement is often contradictory. Many HEIs espouse climate responsibility rhetorically while maintaining high-carbon infrastructures, competitive performance regimes, and investment portfolios misaligned with ecological goals (Helmers et al., 2021; Phelan & Lumb, 2021). Such contradictions create institutional dissonance—a gap between ethical commitment and operational practice—that undermines legitimacy and public trust (Ruiz-Mallén & Heras, 2020).

For higher-education professionals (HEPs)—that is, academics, researchers, and leaders in higher education institutions—this dissonance manifests both ethically and emotionally. Many strongly identify with environmental and social justice values and believe in their integration into the classroom, yet perceive limited agency within bureaucratic or marketized institutional systems for substantive change, raising concerns about student well-being (Skilling et al., 2022). Eco-anxiety among HEPs may, therefore, represent not only planetary concern but also distress arising from the perceived loss of faith in the institutional integrity of social bodies like the government and their own universities. Understanding these emotions provides insight into the social sustainability of knowledge institutions, including their capacity to maintain moral coherence, transparency, and trust amid climate crises.

### 1.2. Eco-Anxiety Beyond Youth: The Professional Gap

Empirical research on eco-anxiety has focused mainly on youth, activists, or general populations (Verplanken et al., 2020; Hickman et al., 2021; Ogunbode et al., 2022). These studies show that climate worry is pervasive and closely tied to perceptions of governmental inadequacy (Ogunbode et al., 2022). However, professionals embedded within sustainability-oriented institutions remain underexplored, despite their pivotal role in climate knowledge production and societal leadership (Pihkala, 2020b). HEPs occupy a dual position as educators of sustainable futures and employees within organisations that may fail to embody them-a moral and occupational paradox that heightens emotional strain (Kelly, 2017; Tollefson, 2021). Early qualitative evidence indicates that sustainability educators frequently experience disillusionment, burnout, and a sense of futility when institutional climate actions are symbolic or performative (Skilling et al., 2022; O’Neill, 2023). Yet systematic, cross-national analysis of eco-anxiety in this group remains undeveloped.

Moreover, existing scholarship rarely considers how eco-anxiety among HEPs may shape organisational capacity, pedagogical practice, or institutions’ ability to provide credible sustainability leadership. Given their central role in modelling climate engagement for students, integrating sustainability across curricula, and shaping public discourse, understanding HEPs’ emotional responses is essential for assessing the relational and ethical dimensions of higher education’s climate governance.

### 1.3. Theoretical Integration: Cognitive Appraisal and Institutional Trust

To address this gap, the present study integrates Cognitive Appraisal Theory (CAT) (Lazarus & Folkman, 1984), which posits that emotions arise from two core appraisals:

(1) the *primary appraisal* of threat significance, and
(2) the *secondary appraisal* of coping capacity.

Applied to climate psychology, these appraisals extend beyond individual coping to encompass collective actors, such as governments and institutions, which have emerged as a focus of climate studies among youth (Hickman et al., 2021). When individuals perceive high environmental threat and experience solastalgia (Albrecht et al., 2007), accompanied by low systemic adequacy, anxiety, and moral distress, they can both intensify. Conversely, transparent and participatory governance can transform anxiety into constructive engagement by reinforcing collective efficacy and trust (Ojala, 2017).

This study proposes an *Institutional Appraisal Model of Eco-Anxiety* (IAMEA), conceptualising eco-anxiety as a relational emotion generated through the interaction between existential threat appraisals and perceptions of institutional authenticity and moral adequacy. In this model, eco-anxiety operates not as a deterministic indicator but as a potential psychosocial signal of perceived alignment or misalignment between institutional rhetoric and climate action. Recognising eco-anxiety in this way reframes it away from an individual pathology to a context-dependent signal of moral injury, institutional strain, and perceived deficits in organisational trustworthiness (Litz et al., 2009).

### 1.4. Study Rationale and Contribution

To examine these dynamics, the study asks: How do higher-education professionals experience and interpret eco-anxiety, and how do perceptions of institutional and governmental adequacy relate to these emotions? To address this question, a global mixed-methods survey was conducted in 2023, integrating quantitative measures of climate anxiety and perceived institutional adequacy with open-ended narrative reflections. This design balances breadth and depth, enabling statistical analysis of associations alongside qualitative insight into lived emotional experience. The study pursues three interrelated aims:

1. To map the prevalence and emotional profile of eco-anxiety within higher education;
2. To examine how perceived adequacy of governmental and institutional responses is associated with climate-related worry; and
3. To interpret narrative accounts of moral and institutional appraisal to illuminate how professionals experience and rationalise sustainability dissonance.

By embedding emotional, ethical, and organisational dimensions within a unified analytic framework, the paper makes three key contributions. First, it situates eco-anxiety within institutional appraisal processes rather than individual psychopathology, emphasising its collective and moral dimensions. Second, it demonstrates correlational rather than causal links, showing that perceived governmental betrayal is a powerful correlate of climate worry even among expert populations, underscoring the role of legitimacy and trust in shaping emotional responses to the climate crisis. Third, it reframes eco-anxiety as a plausible psychosocial lens for examining institutional coherence, revealing how emotions may act as barometers of social sustainability within the higher-education sector. Taken together, these insights advance understanding of the emotional architecture of sustainability transitions and highlight that addressing eco-anxiety through authentic climate leadership and participatory governance is essential for sustaining both psychological well-being and institutional legitimacy in achieving the SDGs.

## 2. Materials and Methods

### 2.1. Study Design and Rationale

This study employed a cross-sectional mixed-methods design, integrating quantitative and qualitative data collected through a single online survey. The approach was designed to capture both the breadth and depth of eco-anxiety as experienced by HEPs—a phenomenon that is simultaneously psychological, moral, and institutional in nature. Quantitative data provided numerical estimates of the prevalence and associations of climate worry, while qualitative narratives offered interpretive insights into its emotional, ethical, and organisational dimensions, presented thematically (Braun & Clarke, 2006). Combining these strands enabled triangulation between measurable correlational associations and lived meanings, enhancing both validity and explanatory richness. A mixed-methods approach was selected because eco-anxiety encompasses affective, cognitive, and relational processes (Pihkala, 2022a), which are insufficiently captured by quantitative scales alone. The integration of numerical and narrative data supports a more holistic account of how professional actors appraise the adequacy of institutions and governments in responding to the climate crisis.

The research was grounded in a critical realist epistemology, recognising that emotions such as eco-anxiety are both *real psychological experiences* and *socially mediated phenomena* (Pihkala, 2020a, 2022b; Hickman et al., 2021). This position assumes that climate-related distress arises not only from environmental threat but also from institutional discourses, governance structures, and trust relationships that shape individual appraisal and collective meaning-making (Pihkala, 2020a). The critical realist stance aligns with the study’s aim to examine eco-anxiety as a relational and ethically grounded emotion—one that reflects the interaction between existential threat appraisal and institutional legitimacy rather than individual pathology. This design is consistent with emerging best practice in sustainability psychology, in which researchers increasingly combine statistical and narrative data to illuminate how professionals and organisations experience climate-related emotions and responsibility (Cunsolo & Ellis, 2018). The study does not infer causality from cross-sectional data; instead, it uses mixed methods to explore patterned associations and their contextualised meanings.

### 2.2. Participants and Sampling

The study targeted HEPs whose primary occupation involved teaching, research, or leadership within recognised higher-education institutions worldwide. This population was selected because HEPs are both contributors to sustainability education and employees within organisations whose practices influence their moral and emotional appraisals of climate responsibility.

Recruitment took place between June and July 2023 through purposive and snowball sampling. Invitations were circulated via international academic and sustainability mailing lists, professional networks (LinkedIn, ResearchGate, and X/Twitter), and direct requests for distribution through institutional channels. Participation was voluntary and anonymous. Inclusion criteria were:

(i) aged 18 years or older;
(ii) currently or recently employed in a higher-education institution;
(iii) English fluency; and
(iv) a professional role primarily involving teaching, research, or leadership.

As open online recruitment was used, a precise response rate could not be calculated. However, participation levels are typical for professional cohort studies in higher education, where the focus is on within-group mechanisms rather than population prevalence (Winfield & Paris, 2024). Given the self-selected recruitment pathways, the sample is not statistically representative of the global HEP population. Instead, it reflects a segment of professionals who were willing to engage with climate-related topics, which may bias the sample toward individuals with stronger concerns or pre-existing interest in sustainability. This limitation is acknowledged explicitly and informs the study’s interpretive rather than prevalence-focused aims.

A total of 556 valid responses met the inclusion criteria, representing participants from 51 countries across six global regions: Europe (41.5%), North America (23.2%), Asia–Pacific (17.8%), Africa (7.4%), Latin America (6.3%), and the Middle East (3.8%). This distribution demonstrates broad geographic coverage, enabling cross-contextual interpretation, but does not support claims of national or regional representativeness. Demographic characteristics—including gender, role type, and years of professional experience—are summarised in Table 1.

The sample size of *N* = 556 provides robust statistical power for the analyses conducted. With α = 0.05, two predictors in the regression model, and an expected small-to-moderate effect size (*f*^2^ ≈ 0.02–0.05), post hoc power analysis indicated >0.95 power to detect significant associations (Faul et al., 2009). This exceeds recommended thresholds for cross-sectional psychological research. Given the correlational design, power is interpreted in terms of detecting associations rather than causal effects. Self-selection likely increased the proportion of sustainability-engaged respondents, as addressed in the Discussion.

### 2.3. Data Collection and Instruments

Data were gathered through an online survey designed to examine the relationships among eco-anxiety, perceptions of collective climate response, and institutional trust. The instrument combined closed- and open-ended items and took approximately 15–20 min to complete. Its structure reflected the mixed-methods rationale: the quantitative component provided structured measures of emotional and cognitive dimensions of climate worry, while the qualitative component offered narrative depth and contextual interpretation.

The primary quantitative instrument was an adapted version of the *Climate Anxiety Scale* developed by Hickman et al. (2021). Originally validated for youth, this scale captures a multidimensional profile encompassing affective responses (e.g., sadness, fear, helplessness), cognitive appraisals (e.g., perceptions of planetary failure and future threat), and perceived societal and political inaction. Because this study focused on HEPs, the original scale was adapted to include occupational and institutional dimensions of climate concern. The adapted instrument comprised four conceptual domains:

1. Emotional responses to climate change (e.g., sadness, anxiety, guilt, anger);
2. Cognitive appraisals of the future (beliefs about ecological collapse and social failure);
3. Functional impacts, such as difficulty concentrating, diminished professional motivation, or withdrawal from work-related activity; and
4. Evaluations of collective response, assessing perceived adequacy, trust, hope, and betrayal toward governments and institutions.

To facilitate direct institutional comparison, a parallel set of items was created by substituting the word “*government*” with “*institution*” in each relevant question. No other linguistic or structural changes were made. This mirroring enabled internally consistent comparisons of systemic adequacy and perceived betrayal at two levels of authority—national (governmental) and proximal (institutional). Reliability analyses demonstrated high internal consistency for both subscales (governmental betrayal α = 0.88; institutional betrayal α = 0.83), exceeding recommended thresholds for psychological measurement reliability.

Alternative measures, such as the *Climate Anxiety Scale* and the *Eco-Anxiety Scale* (Clayton & Karazsia, 2020; Hogg et al., 2021), were reviewed but not selected. While both instruments offer valuable insight into the affective and clinical aspects of climate distress, they primarily emphasise individual symptomatology. They do not adequately capture the institutional and sociopolitical contexts in which climate emotions often arise among professionals working in education, research, and policy. Hickman and colleagues’ instrument was therefore selected for its explicit inclusion of the relational and contextual dimensions of eco-anxiety, including perceived governmental inadequacy, institutional strain, and a sense of abandonment by leadership systems expected to address the climate crisis (Hickman et al., 2021). These sociopolitical dimensions are increasingly recognised as central to understanding eco-anxiety among individuals with strong ethical and professional identification with climate responsibility. Accordingly, the adapted Hickman scale provided the best theoretical and empirical fit for the present study’s aim: to conceptualise eco-anxiety not only as an individual emotional response but as an institutionally mediated psychosocial experience grounded in appraisals of trust, legitimacy, and moral adequacy.

To enhance interpretive richness, the survey included a single optional open-ended question inviting participants to reflect on the emotional and professional implications of climate change: “Do you have any personal reflections, experiences, or thoughts about how climate change affects you emotionally or professionally?” This item was designed to elicit narrative accounts spanning emotional, cognitive, and institutional dimensions not captured by quantitative scales. The inclusion of qualitative data aligns with calls for greater attention to narrative and phenomenological approaches to the study of climate emotions (Cunsolo & Ellis, 2018). In total, 206 participants (37.0%) provided narrative responses. Their diversity illuminated the ways eco-anxiety manifests across affective, cognitive, and institutional domains. Integrating these accounts with the quantitative data enabled a multidimensional analysis of eco-anxiety among HEPs—revealing not only its prevalence and predictors but also how climate distress is shaped by perceived failures of leadership, systemic inaction, and organisational culture.

### 2.4. Data Analysis

Quantitative analyses were conducted using IBM SPSS Statistics (Version 29). Descriptive statistics summarised demographic characteristics and the prevalence of eco-anxiety indicators. Pearson product–moment correlations examined bivariate associations among climate-related worry, perceived adequacy of governmental and institutional responses, and affective indicators (anger, sadness, helplessness, and related emotions). A multiple linear regression model tested whether *perceived adequacy of governmental* and *institutional responses* was associated with eco-anxiety scores. All tests were two-tailed, with *α* = 0.05. Before analysis, assumptions of normality, linearity, and multicollinearity (Variance Inflation Factor < 2) were verified. Heteroskedasticity-consistent (HC3) standard errors were applied to ensure robustness of coefficient estimates. Missing data (<2%) were addressed via listwise deletion. Gender, age, and regional comparisons were beyond the scope of the current analysis and warrant exploration in future work. The final regression model incorporated two primary predictors: perceived governmental and institutional adequacy.

Qualitative data were analysed using Reflexive Thematic Analysis (RTA) (Braun & Clarke, 2006), supported by NVivo 14 software. CAT served as a sensitising framework, linking participants’ emotional expressions to appraisals of climate threat, institutional response adequacy, and collective efficacy (Lazarus & Folkman, 1984). Analysis proceeded through the following iterative stages:

1. *Familiarisation* with the full narrative dataset through repeated readings;
2. *Inductive open coding* of meaningful units reflecting emotional, cognitive, and moral evaluations;
3. *Theme development and refinement*, identifying patterned meanings related to betrayal, legitimacy, and ethical distress; and
4. *Interpretive synthesis*, situating emergent themes within CAT constructs and dimensions of institutional trust.

All coding and analysis were conducted by a single researcher, who engaged in reflexive journaling throughout the process to document analytic decisions, positionality, and emerging interpretations. This reflexive audit trail enhanced transparency and interpretive coherence, consistent with RTA’s emphasis on researcher subjectivity as an analytic resource rather than a bias to be eliminated. Themes were reviewed in light of the quantitative findings to identify convergent, divergent, and complementary insights, enabling a theoretically integrated understanding of how eco-anxiety is socially and institutionally constructed among higher-education professionals.

Integration followed a convergent parallel design, in which quantitative and qualitative data were analysed independently and then merged during interpretation. Triangulation allowed statistical trends to be contextualised through participants’ lived narratives. Quantitative findings provided the prevalence and predictive patterns of eco-anxiety, while qualitative themes explained the moral and institutional appraisals underlying those patterns. To support theoretical integration, this study introduces IAMEA as a concept, which links perceived climate threat, assessments of systemic adequacy, and judgements of institutional legitimacy. This model provided a structured lens for combining quantitative and qualitative insights, clarifying how institutional contexts amplify or mitigate climate-related distress.

### 2.5. Research Ethics and Integrity

The study was reviewed and approved by the Arden University Research Ethics Committee (#P6243). All procedures complied with the Declaration of Helsinki and GDPR/UK Data Protection Act (2018). Electronic informed consent was obtained prior to participation, and respondents were reminded of their right to withdraw at any time before submission. No directly identifying information was requested; however, when disclosed, it was redacted from the reported data, particularly in quotations. Data were stored on encrypted, password-protected university servers accessible only to the research team.

Because several variables could indirectly identify participants, the raw dataset is not publicly available. De-identified data may be shared with bona fide researchers upon reasonable request to the corresponding author, in accordance with the approved data management plan and institutional policies.

During manuscript preparation, OpenAI ChatGPT (GPT-5) was used exclusively for textual editing tasks, such as revising expression and grammar, improving punctuation, and formatting. AI-assisted edits were critically reviewed.

## 3. Results

Findings are presented in a unified narrative that integrates quantitative and qualitative data through the interpretive framework of CAT. This analysis identifies two interdependent processes shaping eco-anxiety among HEPs: the primary appraisal of climate change as a personal and collective threat, and the secondary appraisal of institutional and governmental responses as inadequate or morally compromised. These appraisals interact to generate a distinctive pattern of emotional distress that reflects both existential concern and the erosion of trust in governance and sustainability systems.

### 3.1. Primary Appraisal: Existential Threat and Loss

**Figure 1:**
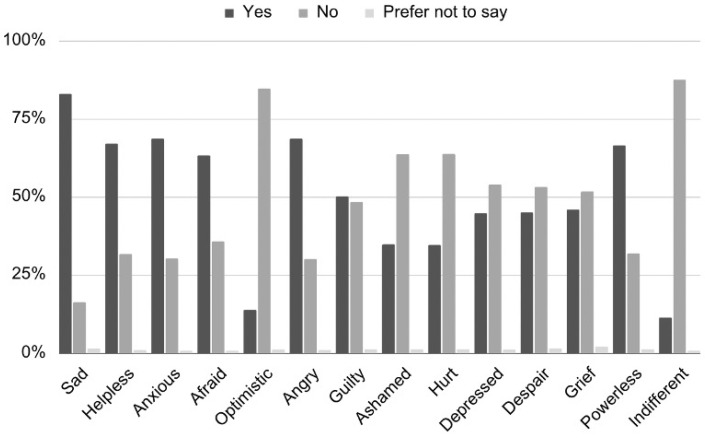
Reported Climate Change-Related Feelings.

Across the total sample (*N* = 556), concern about climate change was high. A total of 81.3% of respondents reported feeling very or extremely worried about climate change, yielding a mean score of 4.26 (SD = 0.93) on a 5-point scale. Negative emotions were widespread: 82.0% reported sadness, 68.7% reported feeling both anxious and angry, 67.1% reported feeling helpless, and 66.5% reported feeling powerless. Fear was reported by 63.3%, and guilt by 50.2%. By contrast, only 14.0% expressed optimism and 11.5% reported indifference (Figure 1).

These emotional profiles reveal a pattern of simultaneous worry, anger, and helplessness consistent with high perceived threat and limited perceived control—the central cognitive profile of anxiety in CAT.

**Figure 2:**
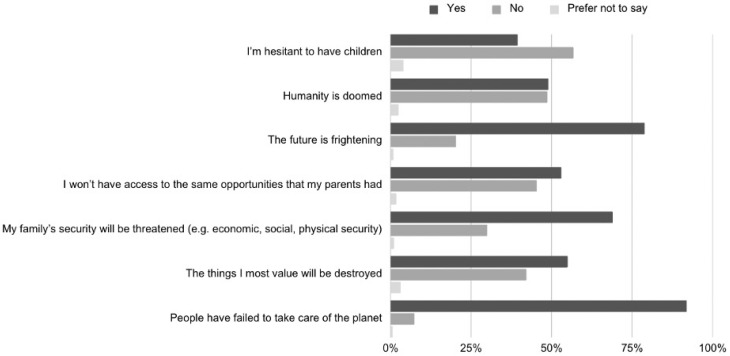
Reported Climate Change-Related Beliefs.

Participants’ cognitive appraisals of climate change reflected similarly bleak perspectives. More than 91.9% agreed with the statement “people have failed to take care of the planet,” and 78.8% described the future as “frightening” (see Figure 2). These beliefs, grounded in emotional appraisal theory, underscore the intersection between existential concern and institutional or systemic trust.

Participants’ narratives conveyed that climate change was experienced not as a distant or abstract risk but as an immediate and escalating threat to well-being, identity, and purpose. Many respondents explicitly linked their professional roles and daily functioning to this ongoing sense of threat. Approximately 30.9% (*N* = 172) reported that climate-related worry adversely affected their daily functioning, including concentration, sleep, and work engagement. One participant wrote:

*I experience eco-anxiety daily. I have tried to act in environmentally sustainable, mindful and compassionate ways for years because of eco-anxiety, which is why I ended up in academia. However, as is often the case, the more you know, the worse it gets. Being in a privileged position of knowing so much about climate science and the plethora of social injustices makes me feel a constant sense of guilt, worry, shame and fear*.

[P392, male, white, Europe and Central Asia, university]

Another respondent described the persistent cognitive load associated with anticipating catastrophe:

*I find it hard to maintain a hopeful positive mood sometimes because everywhere I go in life there are reminders that we are not doing enough. The most visible of these relate to transport so I can’t leave my house without seeing petrol cars driving down the road or when the sky is clear seeing airplane trails in the sky. I find this so depressing and it acts as a continual reminder of the lack of substantial rapid action. I am increasingly thinking about how best to position myself and those I care about for when the inevitable crises come in the future. It is hard to do this preparedness work and contribute to carbon reduction/mitigation while also carrying on with everyday life. It wouldn’t surprise me if one day we ended up with a climate lock down (like we had with COVID) but this still feels a long way off.*

[P58, male, white, Europe and Central Asia, university]

Meanwhile, several respondents provided direct accounts of being personally affected by climate-related events:

*People think the effects of climate change will be felt in the future, but they’re happening now. My family and I were displaced because of a wildfire—we had to live in a hotel for over a month before they’d let us back into the neighbourhood, then we had to move entirely because everything was just burnt and gross. My parents had no power for two weeks and had to replace their roof because of a hurricane… My sister can’t go outside for two months out of the year because she lives in an ever increasingly hot and dry desert… People are in denial that they’re already dealing with the effects of climate change.*

[P34, female, white, North America, regional college]

Others recalled specific events that triggered sustained distress:

*It is not something that affects me every day… It’s more a sense of existential dread, particularly during the Australian 2019/2020 bushfires when it felt [like] the whole world was unravelling. I was in Europe at the time, but could not stop reading the Guardian to see what was happening.*

[P424, female, white, East Asia and the Pacific, vocational university-degree offering institution]

*My health is very poor in heatwaves and I cannot work or go outdoors, so my daily life is badly affected in 30C heat or above. I come from the south of England but cannot imagine moving back there now due to the heat. I used to enjoy summer as a child and teenager, and these last few years with successive heatwaves have found myself grieving for a season that is not there any more. I have turned forty so am probably around halfway through my life—when I think about how I will die I fully expect it to be as an elderly person succumbing to heat stress.*

[P83, non-binary, white, Europe and Central Asia, university]

These reflections illustrate the primary appraisal process in CAT: climate change is interpreted as an immediate, uncontrollable, and morally significant threat. Respondents articulated both cognitive and affective manifestations like guilt, fear, helplessness, and insomnia, which extend beyond abstract concern to tangible disruption in personal and professional functioning. The intensity of these emotions was amplified among those who were most informed about climate science, suggesting that awareness may heighten rather than alleviate distress.

Building on this, participants’ beliefs about human responsibility and the future reflected equally bleak outlooks. Participants reflected on the interconnection between existential concern and systemic trust, revealing that emotional distress is shaped as much by moral reflection as by perceived environmental risk. Participants often situated their anxiety within intergenerational and pedagogical contexts, linking fear to their children and students.

*My child is under 10. I’m terrified for their future. Although things were not great when they were born, it’s really ramped up now.*

[P28, female, white, Europe and Central Asia, university]

Another concluded:

*I feel tremendous anxiety and guilty about having children. They’re doomed.*

[P289, female, white, North America, community college]

As a further example:

*A lot of my emotions on this are not for me but as a parent and on behalf of students I teach.*

[P410, female, white, Europe and Central Asia, university]

Another stated:

*Since I am from the Philippines, an archipelagic nation in the Asia Pacific, greatly affected by disasters (especially extreme weather conditions such as typhoons and El Nino and heat waves), and very vulnerable because of limited resources (Third World country, almost half are living below poverty), the threat of climate change is felt everyday. I see destruction of lives and livelihood, threat in our food source,* etc. *I feel anxious and extremely sad for my young daughter.*

[P372, female, Filipino, East Asia and the Pacific, university]

For educators, eco-anxiety intersected with professional ethics and responsibility for shaping future generations. Some reflected on how they consciously moderated their teaching to sustain hope among students:

*I work in a department (International Development) which is specifically tackling climate change and its impacts with the most vulnerable people in the world… I resist all talk about doom, humanity is going to be wiped out, *etc.*, because people, especially my young students, then feel there is no point in carrying on trying to find solutions… Doom talk is paralysing.*

[P55, white, female, North America, university]

Such reflections illustrate that eco-anxiety among HEPs is not an isolated emotional state but a cognitively and ethically embedded phenomenon. Their distress incorporates anticipatory fear, guilt, and moral concern for others, particularly younger and future generations. These findings reinforce the idea that the primary appraisal of climate change involves both the recognition of threat and the internalisation of moral responsibility, setting the stage for secondary appraisals focused on the perceived adequacy and legitimacy of institutional and governmental responses.

### 3.2. Secondary Appraisal: Institutional Adequacy and Moral Disillusionment

**Figure 3:**
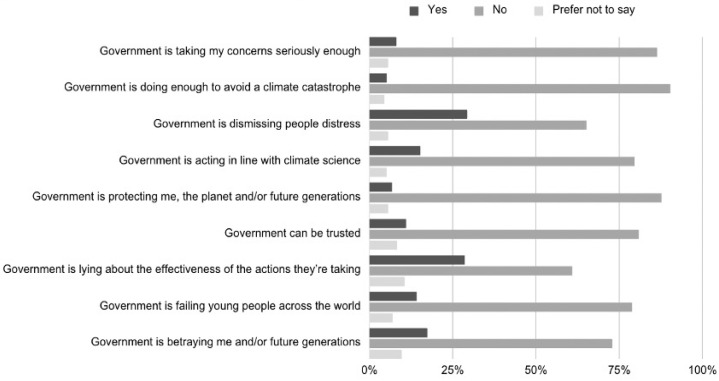
Beliefs About Government Climate Responses.

Participants’ secondary appraisals—that is, their evaluations of the adequacy and authenticity of collective responses—were also bleak. Only 5.2% of respondents believed their government was doing enough to avert a climate catastrophe, while 72.8% reported feeling betrayed by governmental inaction. Mean adequacy scores were 2.01 (SD = 0.95) for government and 2.35 (SD = 1.12) for institutions. Figure 3 captures the key beliefs. Governmental betrayal correlated strongly with climate worry (r = 0.481, *p* < 0.001), and regression analysis confirmed it as a significant predictor (β = 0.481, t = 9.454, *p* < 0.001; adj R^2^ = 0.256). Table 2 captures the correlations.

The multiple regression model (F^(2, 470)^ = 82.374, *p* < 0.001) revealed that government betrayal was a significant predictor of climate worry (β = 0.481, t = 9.454, *p* < 0.001), accounting for 26% of the variance (adj R^2^ = 0.256).

Notably, narrative responses were suffused with emotion. Respondents frequently expressed anger, moral outrage, and despair toward governments and institutions perceived as prioritising economic growth over planetary survival:

*…All the little actions we do (recycling, not using planes, not using the car, growing our own food) are not enough to combat the private jets, the big cars, the overconsumption. The politicians are too busy backstabbing and bickering to make a difference.*

[P28, female, white, Europe and Central Asia, university]

This was echoed by another participant who stated:

*I get very desperate when I see how governments and companies do not do their part to fight climate change. It is proven that it is not the individual who can do that much to prevent climate change. It is the governments and particularly the big companies who don’t give a [care] and just care about money and growth…*

[P120,female, white, Europe and Central Asia, university]

Others emphasised geopolitical inequities and misplaced national priorities:

*We are in a developing country and the concerns are more around poverty, corruption, poor health care, violent crime, gender-based violence… So climate change is quite far down the list of priorities.*

[P198, female, Indian, Sub-Saharan Africa, university]

Another participant reflected:

*…I am incredibly frustrated/angry by the lack of action on the part of our government, both to provide meaningful support on the global stage and to invest the money it has… in building climate resilience and adaptive capacity within the UK.*

[P416, female, white, Europe and Central Asia, university]

This pervasive sense of betrayal appeared to cascade downward, shaping how participants appraised not only governmental authority but also the institutions in which they worked. The same emotional logic—rooted in perceived hypocrisy, moral dissonance, and the prioritisation of economic or reputational interests over ethical responsibility—extended to universities and research organisations. Many respondents suggested that governments and higher education institutions operate within a shared paradigm of *performative sustainability*, in which rhetorical commitments substitute for structural change. In this way, governmental inaction and institutional inertia were experienced as mutually reinforcing, eroding trust and amplifying distress among professionals whose work depends on credible leadership and collective moral purpose.

**Figure 4:**
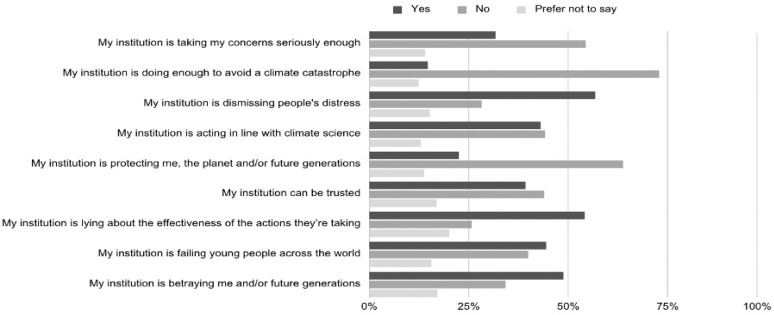
Beliefs About Institutional Climate Responses.

Indeed, at the institutional level, respondents conveyed similar distrust. Although 22.7% felt reassured by their institution’s climate initiatives, 70.9% did not, and 43.7% reported feeling unprotected by institutional policies. While the correlation between institutional betrayal and climate worry was significant (r = 0.365, *p* < 0.001), institutional adequacy was a weaker predictor than governmental betrayal (β = 0.043, *p* = 0.395), suggesting that national-level failures weigh more heavily on affective outcomes. Figure 4 captures the beliefs expressed.

Qualitative data enriched these results. Participants repeatedly characterised university responses as performative, symbolic, or greenwashed:

*One thing that is making me particularly angry is the greenwashing that takes place in higher education. Institutions pretend they are fighting climate change while they are in fact preserving their privileges and not changing a thing.*

[P236, female, declined to identify, Europe and Central Asia, university]

Another observed:

*Universities (such as my employer) should be at the forefront in making the transition to carbon neutral. Unfortunately it is mostly hot air and very little action. There is an enormous gap between rhetoric and action in Higher Education. Unfortunately, the climate and ecological crisis does not respond to nice words, only action. My institution, like most others, is unwilling or unable to do what is needed, whether in terms of its own activities (*e.g.,* dependence on funding from fossil fuel companies, reliance on extensive high-carbon travel) or holding power to account.*

[P206, male, white, Europe and Central Asia, university]

Several expressed resignation that institutional impact was ultimately constrained by governmental inaction:

*I don’t think the institution really has much of an impact on the outcomes of climate change. While they are doing research, it means little if governments don’t take the action that research recommends.*

[P517, male, white, North America, university]

Across both contexts, the emotional vocabulary of respondents—“betrayed,” “manipulated,” “ignored,” “exhausted”—points to moral injury: a profound sense of disillusionment when institutions fail to uphold ethical obligations. This loss of faith often spilled into occupational withdrawal and existential fatigue:

*It has made me rethink the value of the research I do (it now feels pointless and I have more or less given it up).*

[P333, female, white, East Asia and the Pacific, university]

Another stated:

*Thinking about leaving academia to go repair bicycles in the countryside.*

[P316, male, declined to identify, Europe and Central Asia, research institution]

Explanations of daily life added further examples of concern around institutional activities:

*I feel that while there is a lot of rhetoric around sustainability and action in terms of offering sustainability degree courses and centres of excellence across multiple disciplines, student societies, small scale growing on campus* etc. *but at the same operate under growth strategies (student numbers have increased from 6000 to 18,000 since 2000s) which have resulted in large parts of the city being demolished and rebuilt for student housing (owned by university and other businesses) and new development on green belt/previously agricultural land including wetland drainage to create lake landscapes. Further student expansion is planned (anecdotally +30%) focused on international recruit without knowledge/consideration of the environmental impact of international travel. This is echoed in the common practice of sending teams of academics to international conferences and even the environmental costs of such events. While I appreciate and support the diversity this can bring, it is a matter of priorities and maturity and being able to objectively evaluate where the benefit of in-person attendance against costs is significant enough to warrant travel (which should then be carbon offset). For example, sending a team and equipment overseas to showcase an piece of art created for virtual reality—as a digital project/product could this have been achieved without travel? I do not believe that we can continue operating within this ‘business as usual’ mode. We cannot change without change.*

[P114, female, white, Europe and Central Asia, university]

Secondary appraisals, therefore, extended well beyond perceptions of technical competence or procedural adequacy. Participants’ evaluations evolved into judgements regarding the moral legitimacy, accountability, and integrity of the institutions responsible for climate governance, reflecting a broader crisis of trust in these institutions. Respondents did not simply question whether universities or governments were effective; they questioned whether these entities were ethically credible and genuinely committed to collective welfare. The sense that leaders and institutions had violated shared obligations to protect the planet, future generations, and vulnerable communities gave rise to a pervasive experience of moral dissonance.

Within the framework of CAT, this marks a transition from assessing whether something can be done to interrogating whether those in power should be trusted to act at all. Participants’ anxiety was sustained not only by perceptions of uncontrollable threat but also by the conviction that those in positions of authority were acting in their own self-interest, prioritising economic growth and reputational preservation over moral duty. These appraisals transformed eco-anxiety into a moral-cognitive emotion, structured around feelings of betrayal, injustice, and ethical violation. The emotional tone of the dataset reveals that distress was relational, directed toward institutions and governance systems rather than toward the self.

Together, these results demonstrate that eco-anxiety among higher-education professionals arises through the interaction of intense primary appraisals of existential threat and secondary appraisals of institutional and governmental failure. Quantitative models show that both processes independently predict climate-related distress, while qualitative data reveal how they are interwoven in lived experience. Respondents repeatedly rejected pathologising interpretations of their distress, instead framing it as proportionate and ethically rational:

*I feel that every decision I make now is affected by eco anxiety. It’s actually a valid response to an emergency situation so it’s not anxiety for no reason.*

[P346, female, white, Europe and Central Asia, university]

Eco-anxiety thus emerges as an affective index of both environmental degradation and institutional unsustainability—a psychological signal of systemic dysfunction rather than individual maladaptation.

Participants clearly situate eco-anxiety within the realm of social and institutional sustainability, describing a widening gap between the rhetoric of sustainability and the reality of systemic inertia. Within the framework of CAT, this reflects a dual process: a primary appraisal in which climate change is experienced as an immediate and morally significant threat, and a secondary appraisal in which institutions and governments are judged as lacking the capacity, authenticity, or willingness to respond adequately. This disjunction eroded some participants’ sense of belonging and purpose within higher education. For many, eco-anxiety functioned as a form of moral protest, signalling a refusal to normalise institutional hypocrisy or accept insufficient collective action. Their distress emerged not as a pathological response but as an ethically rational reaction to perceived breaches of trust, transparency, and justice. The findings, therefore, position eco-anxiety not merely as a psychological state but as an appraisal-driven affective lens on the erosion of social legitimacy, revealing how emotional well-being, professional identity, and institutional integrity are deeply interdependent within the sustainability agenda.

## 4. Discussion

This study provides an urgently needed examination of eco-anxiety among professionals working in higher education, a group central to sustainability transitions yet underrepresented in research on climate-related mental health. HEPs are not only educators but institutional agents of sustainability: they shape climate knowledge, influence public discourse, and train future leaders. The findings from a global sample of 556 participants across 51 countries demonstrate that HEPs experience climate change as both an epistemic and existential concern—an emotional, moral, and occupational burden embedded in institutional life. Over four-fifths of participants reported being “very” or “extremely” worried about climate change, and nearly one-third described functional impairment linked to this distress. These results extend evidence on the psychological toll of environmental degradation by showing that climate anxiety also functions as an occupational stressor with implications for professional engagement and morale.

The study adds further theoretical and empirical insight by situating eco-anxiety within professional and institutional contexts. While previous scholarship has rightly associated eco-anxiety with perceptions of global threat, uncertainty, and helplessness (Pihkala, 2022a), this research demonstrates that in professional populations, distress is strongly mediated by trust, specifically in systemic responses. Perceived governmental inaction emerged as the strongest correlate of climate worry, aligning with evidence that deficits in efficacy and legitimacy magnify anxiety in young people (Hickman et al., 2021). Institutional responses were evaluated somewhat more favourably but were often described as inconsistent or performative, echoing critiques of “symbolic sustainability” and “greenwashing” within universities. These findings highlight that eco-anxiety in higher education arises not only from fear of ecological collapse but from perceived misalignments between institutional commitments and action, which many respondents interpreted as an abdication of moral responsibility.

Qualitative narratives captured this dynamic vividly. Participants described institutional dissonance—the incongruence between universities’ rhetorical commitments to sustainability and their operational realities. When initiatives were confined to publicity or incremental targets, respondents reported moral exhaustion, loss of purpose, and diminished identification with institutional missions. Similar patterns have been observed among sustainability educators and professionals facing value misalignment and ethical strain (Skilling et al., 2022). This study, therefore, provides additional evidence that eco-anxiety can be experienced as a response to perceived institutional inconsistencies, in which emotional distress reflects moral misalignment and the erosion of trust rather than solely environmental threat appraisal.

The emotional mechanisms underlying these experiences are consistent with CAT. According to Lazarus and Folkman (1984), emotions result from two linked evaluations: the perceived severity of threat (primary appraisal) and perceived adequacy of coping resources (secondary appraisal). For HEPs, climate change is not merely an environmental crisis but also a professional and ethical one. Participants perceived a high existential threat, coupled with low institutional and governmental capacity, which produced anger, helplessness, and moral fatigue. When sustainability rhetoric lacked credibility, cognitive dissonance intensified this distress by exposing contradictions between personal and collective ethics. The proposed IAMEA concept extends this theory by conceptualising eco-anxiety as a relational emotion produced by the interaction between threat appraisal and evaluations of institutional adequacy, authenticity, and legitimacy. Rather than a symptom of individual fragility, eco-anxiety in this model represents an emotion shaped by the perceived adequacy of systemic responses.

Compared with existing eco-anxiety literature, the findings situate this research within and beyond it. Studies of youth populations emphasise anticipatory grief and loss of future security (Hickman et al., 2021). In contrast, this work reveals a more reflexive, institutionally mediated distress among professionals charged with enacting sustainability agendas. It shows that expertise does not inoculate against anxiety; instead, awareness coupled with systemic impotence may be associated with higher levels of distress. By empirically linking trust deficits to emotional outcomes, the study bridges climate psychology and organisational research, responding to calls for interdisciplinary approaches to the social determinants of eco-emotions (Coffey et al., 2021).

Eco-anxiety can thus be interpreted as a multidimensional occupational stressor shaped by leadership culture, ethical coherence, and moral workload. It contributes to the literature on psychosocial risks by identifying climate-related distress as a determinant of well-being and professional engagement within knowledge institutions. Addressing it requires systemic rather than individualised solutions. Universities should recognise the emotional labour inherent in sustainability work and incorporate climate-related well-being into governance and performance frameworks. Evidence-based strategies include participatory decision-making, transparent links between climate commitments and institutional investments, and psychosocial support grounded in collective efficacy and trust-building, rather than solely in individual coping strategies. These findings reinforce that emotional well-being is a structural condition for progress on SDGs 3, 13, and 16.

The findings carry significant implications for policy and practice across both higher education and broader climate governance. For universities, addressing eco-anxiety requires recognising it not as an individual pathology but as a structural signal of how institutional actions are perceived in relation to sustainability commitments. Embedding climate-related well-being into organisational policy through transparent reporting, participatory decision-making, and alignment of financial and research investments with decarbonisation goals can help build trust and credibility. Institutional leaders should cultivate emotionally literate climates that acknowledge climate distress as part of professional life and integrate psychosocial support and reflective dialogue into sustainability strategies.

For policymakers, the results highlight that governmental legitimacy is a critical dimension associated with collective emotional resilience. Transparent, science-based, and justice-oriented climate policies that visibly translate commitment into action can be associated with reduced feelings of betrayal and moral dissonance among professionals and the public alike. Across both domains, cultivating trust, authenticity, and care within governance structures is therefore important for sustaining motivation and engagement in pursuing the SDGs.

Methodological contributions further strengthen the study’s value. Adapting the Hickman Climate Anxiety Scale for professional contexts extends its validity beyond youth samples and confirms the reliability of institutional-level appraisal items (Hickman et al., 2021). The mixed-methods design addresses recent calls to combine quantitative generalisability with qualitative depth in climate-emotion research (Coffey et al., 2021). While the self-selected English-language sample limits generalisability, the study provides an exploratory cross-national portrait of eco-anxiety in higher education, suggesting that concerns about institutional adequacy may be widely shared, though further representative sampling is required. Future longitudinal and cross-cultural work should test IAMEA’s conceptual utility, explore sectoral differences, and examine whether reforms in governance or leadership coincide with changes in perceptions of legitimacy and agency.

Despite these limitations, the study makes an original contribution to sustainability science by conceptualising eco-anxiety as an emotion shaped in part by appraisals of institutional and governmental adequacy situated at the nexus of psychological health, organisational trust, and environmental governance. It provides empirical evidence that perceived systemic inadequacy, primarily governmental, is associated with increased distress and lower perceptions of institutional reliability. This underscores that psychosocial well-being is not peripheral to sustainability transitions but central to their success. Aligning rhetoric with authentic action, embedding care infrastructures within governance, and fostering participatory leadership are, therefore, potential supports for sustaining those who lead climate education and research.

## 5. Conclusions

This paper presents a cross-national analysis of eco-anxiety among HEPs, reframing it as an emotion influenced by both environmental threat appraisal and perceptions of institutional and governmental adequacy at the intersection of climate psychology, organisational trust, and sustainability governance. The findings demonstrate that climate change is experienced within academia not only as a scientific or policy issue but as an existential and professional one. Perceived governmental betrayal emerged as the strongest correlate of climate worry, revealing that distress stems not only from environmental threat but also from the erosion of perceived collective competence. Institutional responses, while somewhat more favourably viewed, were often experienced as symbolic or inconsistent, generating cognitive and moral dissonance.

Reframing eco-anxiety as a phenomenon shaped partly by occupational and relational factors has significant implications for both higher education institutions and policymakers. For universities, climate-related distress should be acknowledged as part of professional experience rather than an individual pathology. Addressing it requires systemic approaches: embedding sustainability throughout governance and operations (SDG 13), ensuring transparent and participatory decision-making (SDG 16), integrating emotionally literate climate education (SDG 4), and providing accessible psychological support (SDG 3). For governments, the findings highlight trust as a critical psychosocial resource. Transparent, accountable, and science-based climate governance is essential for supporting public confidence and engagement.

Ultimately, eco-anxiety among educators and researchers should be understood as a multidimensional emotional response that reflects tensions between knowledge and action, responsibility and inertia. Building emotionally resilient, ethically coherent institutions is thus important to sustainable development. To safeguard both planetary and psychological well-being, universities and governments may need to invest not only in decarbonisation but in infrastructures of care, trust, and accountability that sustain meaningful engagement with the climate crisis.

## Figures and Tables

**Table 1:** Participant characteristics.

Variable	Category	%
Gender	Female	53.8
	Male	43.1
	Other/prefer not to say	3.1
Age group	18–34	15.6
	35–44	30.8
	45–54	22.8
	55+	30.8
Ethnicity	White	67.6
	Black	15.6
	Asian	4.0
	Mixed/Other	12.8
Neurodiverse	Yes	17.5
Disabled	Yes	8.8

**Table 2:** Correlation Matrix for the Study Variables.

	1	2	3	4	5	6	7	8	9	10	11	12	13	14	15	16
1: Worried about CC	1	0.648 **	0.596 **	0.303 **	0.315 **	0.481 **	−0.138 **	−0.130 **	−0.171 **	−0.165 **	0.222 **	0.348 **	0.069	−0.120 **	−0.122 **	−0.070
2: Negative feelings	0.648 **	1	0.774 **	0.453 **	0.407 **	0.614 **	−0.205 **	−0.194 **	−0.189 **	−0.215 **	0.281 **	0.453 **	0.176 **	−0.144 **	−0.106 *	−0.090 *
3: Negative thoughts	0.596 **	0.774 **	1	0.549 **	0.351 **	0.593 **	−0.153 **	−0.136 **	−0.132 **	−0.165 **	0.329 **	0.486 **	0.212 **	−0.124 **	−0.134 **	−0.093 *
4: Negative functional impact	0.303 **	0.453 **	0.549 **	1	0.168 **	0.407 **	−0.019	0.002	−0.048	−0.042	0.193 **	0.359 **	0.073	−0.018	−0.044	−0.005
5: Negative beliefs (government)	0.315 **	0.407 **	0.351 **	0.168 **	1	0.507 **	−0.503 **	−0.454 **	−0.499 **	−0.512 **	0.316 **	0.208 **	0.174 **	−0.120 *	−0.146 **	−0.138 **
6: Feeling betrayed (government)	0.481 **	0.614 **	0.593 **	0.407 **	0.507 **	1	−0.285 **	−0.265 **	−0.270 **	−0.264 **	0.284 **	0.626 **	0.141 **	−0.002	−0.026	0.026
7: Feeling reassured (government)	−0.138 **	−0.205 **	−0.153 **	−0.019	−0.503 **	−0.285 **	1	0.433 **	0.523 **	0.553 **	−0.197 **	−0.107 *	−0.253 **	0.151 **	0.204 **	0.148 **
8: Feeling hopeful (government)	−0.130 **	−0.194 **	−0.136 **	0.002	−0.454 **	−0.265 **	0.433 **	1	0.613 **	0.605 **	−0.221 **	−0.133 **	−0.234 **	0.272 **	0.219 **	0.213 **
9 Feeling protected (government)	−0.171 **	−0.189 **	−0.132 **	−0.048	−0.499 **	−0.270 **	0.523 **	0.613 **	1	0.672 **	−0.222 **	−0.072	−0.280 **	0.246 **	0.279 **	0.233 **
10: Feeling valued (government)	−0.165 **	−0.215 **	−0.165 **	−0.042	−0.512 **	−0.264 **	0.553 **	0.605 **	0.672 **	1	−0.185 **	−0.077	−0.261 **	0.281 **	0.264 **	0.259 **
11: Negative beliefs (institution)	0.222 **	0.281 **	0.329 **	0.193 **	0.316 **	0.284 **	−0.197 **	−0.221 **	−0.222 **	−0.185 **	1	0.648 **	0.597 **	−0.564 **	−0.570 **	−0.521 **
12: Feeling betrayed (institution)	0.348 **	0.453 **	0.486 **	0.359 **	0.208 **	0.626 **	−0.107 *	−0.133 **	−0.072	−0.077	0.648 **	1	0.390 **	−0.289 **	−0.226 **	−0.306 **
13: Feeling reassured (institution)	0.069	0.176 **	0.212 **	0.073	0.174 **	0.141 **	−0.253 **	−0.234 **	−0.280 **	−0.261 **	0.597 **	0.390 **	1	−0.357 **	−0.280 **	−0.328 **
14: Feeling hopeful (institution)	−0.120 **	−0.144 **	−0.124 **	−0.018	−0.120 *	−0.002	0.151 **	0.272 **	0.246 **	0.281 **	−0.564 **	−0.289 **	−0.357 **	1	0.816 **	0.790 **
15: Feeling protected (institution)	−0.122 **	−0.106 *	−0.134 **	−0.044	−0.146 **	−0.026	0.204 **	0.219 **	0.279 **	0.264 **	−0.570 **	−0.226 **	−0.280 **	0.816 **	1	0.718 **
16: Feeling valued (institution)	−0.070	−0.090 *	−0.093 *	−0.005	−0.138 **	0.026	0.148 **	0.213 **	0.233 **	0.259 **	−0.521 **	−0.306 **	−0.328 **	0.790 **	0.718 **	1

** Correlation is significant at the 0.01 level (2-tailed). * Correlation is significant at the 0.05 level (2-tailed).

## Data Availability

Raw data are not publicly available because some responses could indirectly identify participants, particularly those in small states or small island nations with limited academic and researcher populations. De-identified data outputs supporting the findings are available from the corresponding author on reasonable request, in accordance with the ethics committee’s requirements.

## Abbreviations

The following abbreviations are used in this manuscript:

CAT	Higher Education Institution
HEPs	Higher Education Professionals
SDGs	Sustainable Development Goals
